# Ultrasound-guided transversus abdominis plane block using ropivacaine and different doses of perineural dexmedetomidine for analgesia after cesarean section: a randomized controlled clinical trial

**DOI:** 10.3389/fphar.2026.1846214

**Published:** 2026-07-24

**Authors:** Zhenglian Gao, Ming Li, Shihai Mu, Fayu Dong, Bangjian Zhang, Chang Yang, Yixin Liu

**Affiliations:** 1 Department of Anesthesiology, Panzhihua Central Hospital, Panzhihua, Sichuan, China; 2 Department of Nuclear Medicine, Panzhihua Central Hospital, Panzhihua, Sichuan, China

**Keywords:** analgesia, caesarean section, dexmedetomidine, ropivacaine, transversus abdominis plane block

## Abstract

**Background:**

Dexmedetomidine is frequently combined with ropivacaine in ultrasound-guided transversus abdominis plane (TAP) block for analgesia after cesarean section (CS), but is associated with adverse effects. The ideal dose of dexmedetomidine as local anesthetic adjuvant has not been determined. This study aimed to explore the dose of perineural dexmedetomidine for ensuring analgesic efficacy while minimizing adverse effects.

**Methods:**

Patients undergoing CS under combined spinal-epidural anesthesia were randomly allocated to five groups: DR (0.00 μg/kg dexmedetomidine), DR1 (0.25 μg/kg), DR2 (0.50 μg/kg), DR3 (0.75 μg/kg), and DR4 (1.00 μg/kg), all combined with ropivacaine for TAP block. The co-primary outcomes were time to first rescue analgesia and total consumption of sufentanil at 48 h. The secondary outcomes included visual analogue scale (VAS) scores; Ramsay Sedation Scale scores; patient satisfaction scores; time to first ambulation; length of hospital stay; and postoperative complications. Success was defined as achieving statistical significance for both primary endpoints.

**Results:**

Among 150 enrolled patients, 12 were excluded for protocol violations, leaving 138 participants in the final analysis. With increasing doses of dexmedetomidine, time to first rescue analgesia was significantly prolonged (from 5.42 ± 2.29 h to 11.26 ± 2.48 h; *P* < 0.001), and total consumption of sufentanil at 48 h was markedly reduced (from 86.62 ± 16.58 µg to 47.44 ± 15.41 µg; *P* < 0.001), indicating a dose-dependent effect. In pairwise comparisons, groups DR3 and DR4 were both superior to lower-dose groups in prolonging time to first rescue analgesia, reducing total consumption of sufentanil, and shortening time to first ambulation, with no significant difference between DR3 and DR4. With increasing dexmedetomidine doses, the incidence of sinus bradycardia (*P* < 0.001) increased, while the incidence of postoperative nausea and vomiting (*P* < 0.001) decreased. No significant differences were observed among the five groups for VAS pain scores (group × time interaction effects), length of hospital stay, incidence of hypotension, and patient satisfaction scores.

**Conclusion:**

Dexmedetomidine as an adjuvant to ropivacaine in ultrasound-guided TAP block for analgesia after CS, demonstrated a dose-dependent effect. 0.75 μg/kg dexmedetomidine appeared to provide the most favorable balance between analgesic efficacy and safety.

**Clinical Trial Registration:**

https://www.chictr.org.cn, identifier ChiCTR2300068207.

## Introduction

The use of cesarean section (CS) has steadily increased worldwide and will continue increasing over the current decade (Boerma et al., 2018; Betran et al., 2021), and postoperative analgesia is not addressed adequately. Post-cesarean pain within 24 h ranks ninth among 179 surgical procedures (Gerbershagen et al., 2013), and the associated abdominal wall incision and soft tissue dissection may result in moderate to severe postoperative pain, which can adversely affect early ambulation and breastfeeding (Chou et al., 2016). Compared to other surgeries, pain management following CS presents unique demands, particularly during the first 24 h postoperatively, aiming to promote early ambulation, shorten recovery periods, and facilitate optimal maternal-neonatal bonding (Zandomenico et al., 2022). Pain relief must be both rapid and effective, with minimal adverse effects on the mother and the infant. Currently, analgesic methods following CS mainly include patient-controlled intravenous analgesia (PCIA), patient-controlled epidural analgesia, and regional nerve blocks (Sultan et al., 2021). However, these methods have certain limitations. For instance, epidural analgesia is commonly associated with numbness and weakness in the lower limbs, which can delay early ambulation and increase the risk of thrombotic events. Additionally, since opioids are the primary agents used in PCIA, there are opioid-related complications, including sedation, postoperative nausea and vomiting (PONV), pruritus, respiratory depression, and urinary retention. Therefore, it is important to explore safer and longer-acting postoperative analgesic techniques.

The transversus abdominis plane (TAP) block is a regional anesthetic technique that alleviates somatic pain by administering local anesthetic between the internal oblique and transversus abdominis muscles, thereby blocking neural afferents within the transversus abdominis plane (Mallan et al., 2019). Ultrasound-guided TAP block is easy to perform with a good safety profile, providing well-defined analgesia in the T6-L1 dermatomal area over the anterior abdominal wall (Rafi, 2001; Mallan et al., 2019). Currently, the ultrasound-guided TAP block is the most widely used nerve block technique for postoperative analgesia after CS (Mansour et al., 2025; Wang et al., 2026). Studies have demonstrated that ultrasound-guided TAP block not only significantly improves postoperative analgesia and reduces opioid consumption and adverse effects after CS (Nedeljkovic et al., 2020; Sultan et al., 2020; El-Boghdadly et al., 2021), but may also facilitate early mobilization, gastrointestinal recovery, and patient satisfaction (Arslan et al., 2023). However, a key limitation of TAP block lies in their relatively short duration of analgesia, which stems from the short duration of action of the local anesthetics utilized in this technique. To address this challenge, identifying an adjuvant to the local anesthetic that can prolong the duration of the TAP block has generated significant interest.

Local anesthetic adjuvants, such as dexamethasone, dexmedetomidine, midazolam, and ketamine, can reduce local anesthetic dose requirements and associated adverse effects, shorten onset time, prolong analgesic duration, and improve analgesia quality (Prabhakar et al., 2019; Küçüksaraç et al., 2024). Dexmedetomidine is a highly selective α_2_-adrenergic receptor agonist characterized by sedation, analgesia, anxiolysis, sympathetic inhibition, mild respiratory depression, and hemodynamic stability. Numerous studies have demonstrated that dexmedetomidine used in peripheral nerve blocks can shorten the onset time of anesthesia and prolong the duration of sensory and motor blocks, making it one of the most widely used and ideal adjuvants for local anesthetics (Xiong et al., 2022; Chen et al., 2023). In addition, previous clinical studies have highlighted that dexmedetomidine combined with ropivacaine for TAP block is safe and effective for postoperative analgesia after CS (Qian et al., 2020; Jamshidi et al., 2024; Nag et al., 2024). However, the dose of perineural dexmedetomidine varies widely across studies (0.25–2.00 μg/kg). Higher doses (e.g., above 1.00 μg/kg) are associated with increased risks of bradycardia, hypotension, and excessive sedation, without providing additional analgesic benefit (Raof et al., 2017; Zeng et al., 2020). Currently, the dexmedetomidine dose that balances analgesic efficacy and safety when used as an adjuvant to ropivacaine in TAP block remains to be determined.

We therefore designed a study to evaluate the safety and efficacy of different doses of dexmedetomidine combined with ropivacaine administered via ultrasound-guided TAP block for postoperative analgesia after CS.

## Methods

### Patients

This prospective, randomized, double-blinded, controlled clinical trial was approved by the Research Ethics Committee of Panzhihua Central Hospital (Pan-Ke-Lun-Shen-Zi [2023-001]) and registered on the Chinese Clinical Trial Registry (CHICTR; www.chictr.org.cn; registration no. ChiCTR2300068207) before enrollment of the first patient. All patients willing to participate in the study provided written informed consent. This study followed the CONSORT guidelines for randomized trials. It should be noted that perineural administration of dexmedetomidine remains an off-label use. All patients were fully informed of the off-label nature of the intervention and its potential risks and benefits prior to providing consent.

The inclusion criteria were as follows: women aged 19–45 years with normal, uncomplicated singleton pregnancies; a body mass index (BMI) 18–30 kg/m^2^; gestational age of 37 weeks or more; the American Society of Anesthesiologists (ASA) physical status I-II; planned combined spinal-epidural anesthesia (CSEA); and the ability and willingness to use the visual analogue scale (VAS) to report pain. Exclusion criteria were patient refusal; allergy to study medications; systemic or puncture site infection; coagulopathy; chronic use of opioids, sedatives, antidepressants, or monoamine oxidase inhibitors; high-risk pregnancy (including severe diabetes or hypertension, serious cardiovascular diseases [heart failure, severe arrhythmias, sinus bradycardia, or conduction block], severe respiratory disorders [e.g., obstructive sleep apnea], hepatorenal insufficiency, mental disorders or nervous system diseases, serious complications in the previous pregnancy, and other potential pregnancy-related complications); acute emergency; language communication disorder; height <145 cm; and follow-up failure. All patients underwent CS in the Panzhihua Central Hospital.

From 15 February 2023 to 26 September 2024, all patients were screened for eligibility. The conclusion date for patient recruitment was extended from the originally planned January 2024 to October 2024 due to a decrease in CS and a substantial number of exclusions. Importantly, this extension was approved by our Research Ethics Committee prior to the original deadline (Pan-Ke-Lun-Shen-Zi [2024-003]).

### Study design and randomization

After written informed consent, all patients were randomly assigned to one of the five groups according to a computer-generated randomization table.

- Group DR: Received ultrasound-guided bilateral TAP block with addition of dexmedetomidine 0.00 μg/kg to 0.25% ropivacaine solution (20 mL each side)
- Group DR1: Received ultrasound-guided bilateral TAP block with addition of dexmedetomidine 0.25 μg/kg to 0.25% ropivacaine solution (20 mL each side)
- Group DR2: Received ultrasound-guided bilateral TAP block with addition of dexmedetomidine 0.50 μg/kg to 0.25% ropivacaine solution (20 mL each side)
- Group DR3: Received ultrasound-guided bilateral TAP block with addition of dexmedetomidine 0.75 μg/kg to 0.25% ropivacaine solution (20 mL each side)
- Group DR4: Received ultrasound-guided bilateral TAP block with addition of dexmedetomidine 1.00 μg/kg to 0.25% ropivacaine solution (20 mL each side)

Upon enrollment, the anesthesiologist who was not involved in the anesthetic management of the patients or the collection of data delivered a blinded syringe, standardized in a volume of 20 mL, labeled only with the study name and subject number. Blinding was maintained for all clinicians, patients, and research staff until all patients completed the study protocol. If severe adverse reactions occurred, the subject would be withdrawn from the trial and receive active treatment.

### Anesthesia and TAP block procedure

In the operating room, all patients were monitored by noninvasive arterial blood pressure monitoring, electrocardiogram, and peripheral pulse oxygen saturation. All the CS were performed under CSEA, which was initiated in the lateral decubitus position at the L2-3 or L3-4 intervertebral spaces with 15 mg of 0.5% ropivacaine heavy. Although an epidural catheter was placed in all patients, no additional local anesthetic was administered through it because the spinal block alone provided sufficient surgical anesthesia in all cases. After the induction of anesthesia, patients were immediately placed in the supine position with 15°–20° left uterine displacement, and supplemental oxygen was delivered through a facemask at 5 L/min. Surgery was allowed to proceed after T4-6 sensory blockade had been established. Intravenous crystalloids and phenylephrine were administered as needed to treat hypotension. At the end of surgery, all patients received an ultrasound-guided bilateral TAP block performed by an experienced anesthesiologist, according to the standard protocol and their study group allocation.

Under aseptic precautions, a high-frequency (5–12 MHz) linear array ultrasound probe (SonoSite, Bothell, Washington) was placed transversely on the anterolateral abdominal wall between the iliac crest and the costal margin for scanning. The three muscle layers—the external oblique, internal oblique, and transversus abdominis—were identified, and the TAP was identified at the level of the umbilicus along the midaxillary line. Ultrasound-guided TAP block was performed by using a 22-gauge 100 mm peripheral nerve block needle (Pajunk GmbH) with in plane approach. When the needle tip was advanced into the fascial plane between the internal oblique and transversus abdominis muscles, 1 mL of saline was injected following negative aspiration to ensure accurate drug diffusion in the appropriate position. Subsequently, 20 mL of local anesthetic solution was injected. The procedure was then repeated on the other side to complete bilateral TAP block. All patients were received postoperative analgesia via the PCIA pump (150 mL of 1 μg/mL sufentanil; no initial dose; no background dose; bolus of 3 mL per demand; locking time of 10 min; maximum dose of 20 mL/h).

### Data collection

A member of the research team, unaware of group allocation, collected all data. Patient characteristics, including age, height, weight, BMI, ASA scores, surgical type (elective or emergency), gestational age, primiparous or multiparous, previous CS, mean arterial pressure (MAP), heart rate (HR), preoperative complications, duration of surgery, and blood loss, were recorded. The primary outcomes in this study were time to first rescue analgesia (hours), that is, the time to first requested PCIA bolus and total consumption of sufentanil within 48 h after surgery (µg). The secondary outcomes were VAS scores at 2, 4, 6, 12, 24, and 48 h postoperatively at rest and with movement; Ramsay Sedation Scale (RSS) scores at 2, 4, 6, 12, 24, and 48 h after surgery; patient satisfaction scores; time to first ambulation (hours); length of hospital stay (hours); and postoperative complications (including hypoxemia, hypotension, sinus bradycardia, respiratory depression, pruritus, neurotoxicity, and PONV). Since the RSS scores of all participants at each time point were 2 or 3, this variable was not included in the final analysis.

### Statistical analysis

The study was designed with co-primary endpoints: the time to first rescue analgesia (hours) and the total consumption of sufentanil within 48 h after surgery (µg). Success required statistical significance for both primary endpoints. Accordingly, sample size calculations were performed separately for each primary endpoint, and the larger required sample size was adopted for the trial. Based on previous studies (Schnabel et al., 2018; Laigaard et al., 2021; Nag et al., 2024) and our pilot data, mean differences of 5.0 h (standard deviation [SD], 3.0 h) for the time to first rescue analgesia and 35 µg (SD, 20 µg) for the total consumption of sufentanil at 48 h were assumed. These differences were considered clinically meaningful, as a 5.0-h prolongation of analgesia can reduce the need for rescue analgesia during the first postoperative night, and a 35-µg reduction in opioid consumption may decrease the incidence of opioid-related adverse events. Sample size was calculated using multiple comparisons corrections (Tukey-Kramer method) in PASS software version 23.0.2 (NCSS, LLC. Kaysville, Utah, United States), and assuming a 2-sided significance level of 5% and a power of 90%. The analysis indicated that 27 participants per group were required for time to first rescue analgesia and 25 participants per group for total consumption of sufentanil at 48 h. Consequently, 27 participants per group were adopted, which was increased to 30 participants to accommodate a 10% drop-out rate.

The normality of continuous variables was assessed using the Shapiro-Wilk test, and the homogeneity of variances was assessed using Levene’s test. Variables that were normally distributed and exhibited homogeneous variances were expressed as mean ± SD, and comparisons among multiple groups were conducted using one-way analysis of variance (ANOVA). If a significant overall difference was detected, Tukey’s honestly significant difference (HSD) test was applied to *post hoc* pairwise comparisons, and linear and quadratic trend analyses were conducted. For variables with unequal variances or non-normal distribution, data were presented as median (interquartile range, IQR), and group comparisons were performed using the Kruskal–Wallis H test, followed by Dunn’s test for *post hoc* pairwise comparisons with Bonferroni correction. Categorical variables were expressed as numbers (%). Group comparisons were performed using Pearson’s chi-squared test or Fisher’s exact test as appropriate, and dose-response relationships were assessed using Logistic regression analysis. Repeated measures data was evaluated by repeated measures ANOVA or linear mixed-effects model (LMM). The Bonferroni test adjustment was applied for multiple comparisons. A two-tailed *P* value <0.05 was considered statistically significant. All analyses were performed using R software version 4.4.2 (R Foundation for Statistical Computing, Austria).

## Results

**FIGURE 1:**
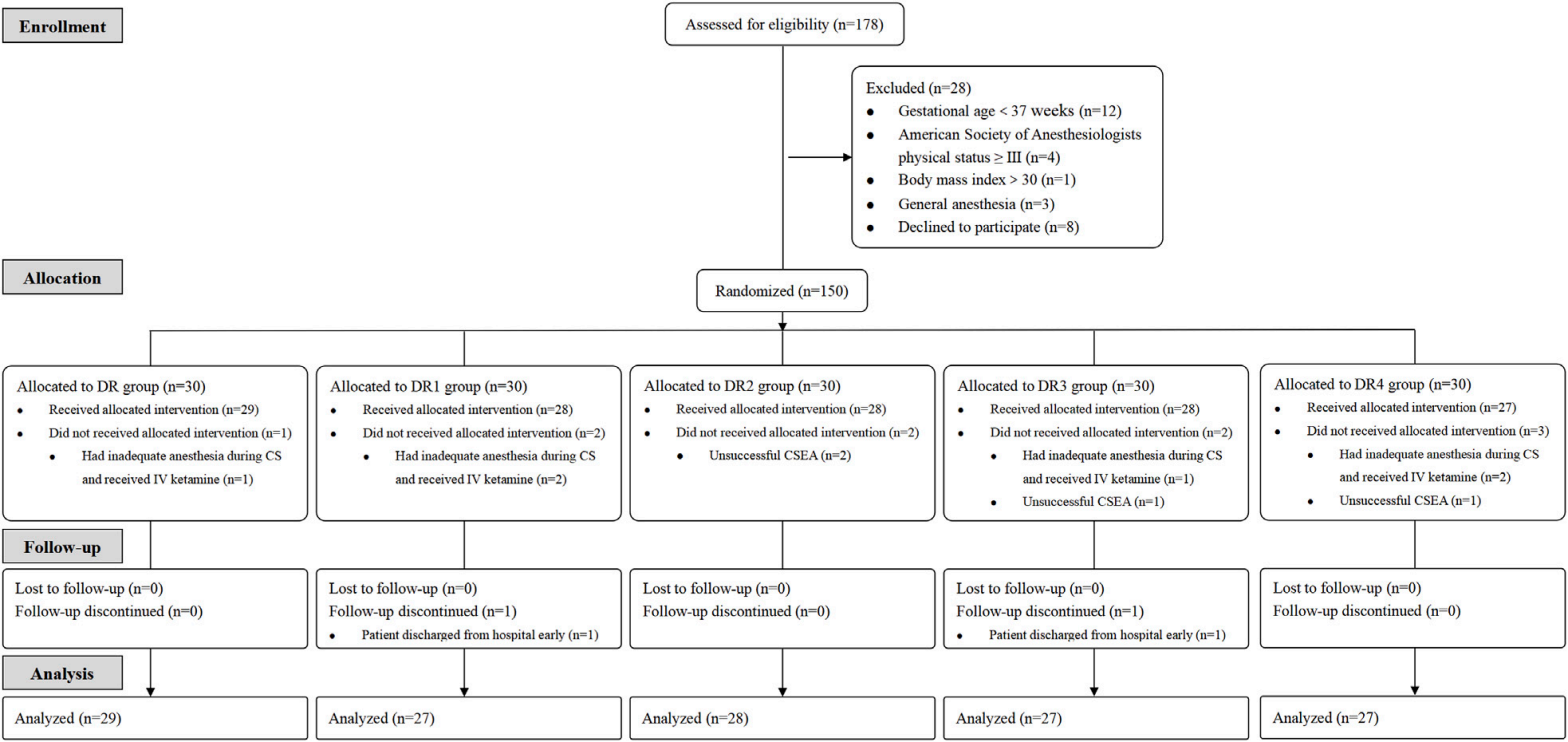
CONSORT flowchart. CS = cesarean section; CSEA = combined spinal-epidural anesthesia.

From 15 February 2023 to 26 September 2024, 178 patients were screened for eligibility, and a total of 150 were enrolled and 138 completed the study (Figure 1). Among the twelve patients who discontinued trial participation due to protocol violations, four did not receive the study medication due to failed CSEA, six received intravenous ketamine intraoperatively due to inadequate anesthesia, and two were lost to follow-up due to early discharge, as shown in Figure 1. All remaining patients successfully completed data collection procedures. Participant demographics and baseline characteristics were balanced across study groups (Table 1).

**TABLE 1:** Baseline characteristics.

Variable	Group DR (n = 29)	Group DR1 (n = 27)	Group DR2 (n = 28)	Group DR3 (n = 27)	Group DR4 (n = 27)	P Value
Age (years)	29.41 ± 4.64	30.11 ± 4.64	30.89 ± 5.09	30.07 ± 4.30	31.78 ± 4.73	0.391
Height (cm)	160.34 ± 4.99	160.67 ± 4.54	158.96 ± 4.67	159.33 ± 3.85	157.85 ± 5.23	0.182
Weight (kg)	67.15 ± 7.36	68.13 ± 7.05	63.86 ± 5.99	66.30 ± 6.85	64.13 ± 7.35	0.099
BMI (kg/m^2^)	26.10 ± 2.49	26.34 ± 2.07	25.26 ± 2.09	26.08 ± 2.32	25.70 ± 2.48	0.446
MAP (mmHg)	87.14 ± 5.94	87.41 ± 6.43	88.46 ± 8.90	88.59 ± 8.33	88.96 ± 7.78	0.872
HR (bpm)	86.83 ± 13.51	90.26 ± 10.64	86.68 ± 9.70	92.07 ± 11.80	91.26 ± 12.08	0.272
ASA score, I/II	​	​	​	​	​	0.587
I	8 (27.6)	5 (18.5)	6 (21.4)	7 (25.9)	10 (37.0)	​
II	21 (72.4)	22 (81.5)	22 (78.6)	20 (74.1)	17 (63.0)	​
Surgical type	​	​	​	​	​	0.458
Elective	8 (27.6)	7 (25.9)	7 (25.0)	3 (11.1)	4 (14.8)	​
Emergency	21 (72.4)	20 (74.1)	21 (75.0)	24 (88.9)	23 (85.2)	​
Preoperative complications	18 (62.1)	12 (44.4)	14 (50.0)	16 (59.3)	8 (29.6)	0.116
Primiparous	14 (48.3)	8 (29.6)	10 (35.7)	16 (59.3)	9 (33.3)	0.149
Previous CS	11 (37.9)	18 (66.7)	13 (46.4)	9 (33.3)	13 (48.1)	0.127
Gestational age (weeks)	39.00 (38.00, 40.00)	39.00 (38.00, 39.00)	39.00 (39.00, 39.00)	39.00 (39.00, 39.50)	39.00 (38.50, 40.00)	0.747
Surgical duration (minutes)	40.00 (34.00, 45.00)	41.00 (36.50, 48.00)	41.00 (37.75, 50.00)	39.00 (34.00, 49.00)	40.00 (35.00, 45.50)	0.569
Blood loss (mL)	400.00 (300.00, 400.00)	400.00 (300.00, 400.00)	375.00 (300.00, 400.00)	400.00 (300.00, 400.00)	400.00 (300.00, 400.00)	0.532

Values were presented as mean ± standard deviation, median (interquartile range), or number (%).

ASA, american society of anesthesiologists physical; BMI, body mass index; CS, cesarean section; HR, heart rate; MAP, mean arterial pressure.

## Primary outcomes

### Time to first rescue analgesia

The time to first rescue analgesia was 5.42 ± 2.29 h in group DR, 6.60 ± 2.58 h in group DR1, 7.88 ± 2.64 h in group DR2, 10.85 ± 2.47 h in group DR3, and 11.26 ± 2.48 h in group DR4. The overall difference among groups was statistically significant (*P* < 0.001) (Table 2). Pairwise comparisons (Table 3) showed that compared with group DR, time to first rescue analgesia was prolonged by 2.45 h (95% confidence interval [CI]: 0.63 to 4.28; *P* = 0.003) in group DR2, by 5.42 h (95% CI: 3.58 to 7.27; *P* < 0.001) in group DR3, and by 5.83 h (95% CI: 3.99 to 7.68; *P* < 0.001) in group DR4. Compared with group DR1, groups DR3 and DR4 prolonged time to first rescue analgesia by 4.25 h (95% CI: 2.37 to 6.12; *P* < 0.001) and 4.66 h (95% CI: 2.78 to 6.53; *P* < 0.001), respectively. Compared with group DR2, groups DR3 and DR4 prolonged time to first rescue analgesia by 2.97 h (95% CI: 1.11 to 4.83; *P* < 0.001) and 3.38 h (95% CI: 1.52 to 5.24; *P* < 0.001), respectively. No other comparisons were significant.

**TABLE 2:** Results of one-way ANOVA for the primary outcomes.

Group	Time to first rescue analgesia (hours)		Total consumption of sufentanil at 48 h (μg)	
	Mean ± standard deviation	P Value	Mean ± standard deviation	P Value
DR	5.42 ± 2.29	<0.001	86.62 ± 16.58	<0.001
DR1	6.60 ± 2.58		82.67 ± 15.08	
DR2	7.88 ± 2.64		71.43 ± 17.28	
DR3	10.85 ± 2.47		55.00 ± 16.30	
DR4	11.26 ± 2.48		47.44 ± 15.41	

**TABLE 3:** Results of Tukey’s HSD test for the primary outcomes.

Comparison	Time to first rescue analgesia (hours)		Total consumption of sufentanil at 48 h (μg)	
	Mean difference (95% confidence interval)	P.adj	Mean difference (95% confidence interval)	P.adj
DR vs. DR1	1.18 [-0.67, 3.02]	0.399	−3.95 [-15.91, 8.00]	0.891
DR vs. DR2	2.45 [0.63, 4.28]	0.003	−15.19 [-27.04, −3.35]	0.005
DR vs. DR3	5.42 [3.58, 7.27]	<0.001	−31.62 [-43.58, −19.66]	<0.001
DR vs. DR4	5.83 [3.99, 7.68]	<0.001	−39.18 [-51.13, −27.22]	<0.001
DR1 vs. DR2	1.28 [-0.58, 3.14]	0.322	−11.24 [-23.30, 0.82]	0.080
DR1 vs. DR3	4.25 [2.37, 6.12]	<0.001	−27.67 [-39.83, −15.50]	<0.001
DR1 vs. DR4	4.66 [2.78, 6.53]	<0.001	−35.22 [-47.39, −23.05]	<0.001
DR2 vs. DR3	2.97 [1.11, 4.83]	<0.001	−16.43 [-28.49, −4.37]	0.002
DR2 vs. DR4	3.38 [1.52, 5.24]	<0.001	−23.98 [-36.04, −11.93]	<0.001
DR3 vs. DR4	0.41 [-1.47, 2.28]	0.975	−7.56 [-19.72, 4.61]	0.427

P values from Tukey’s HSD test were denoted as P. adj, values with P. adj <0.05 were considered statistically significant.

**FIGURE 2:**
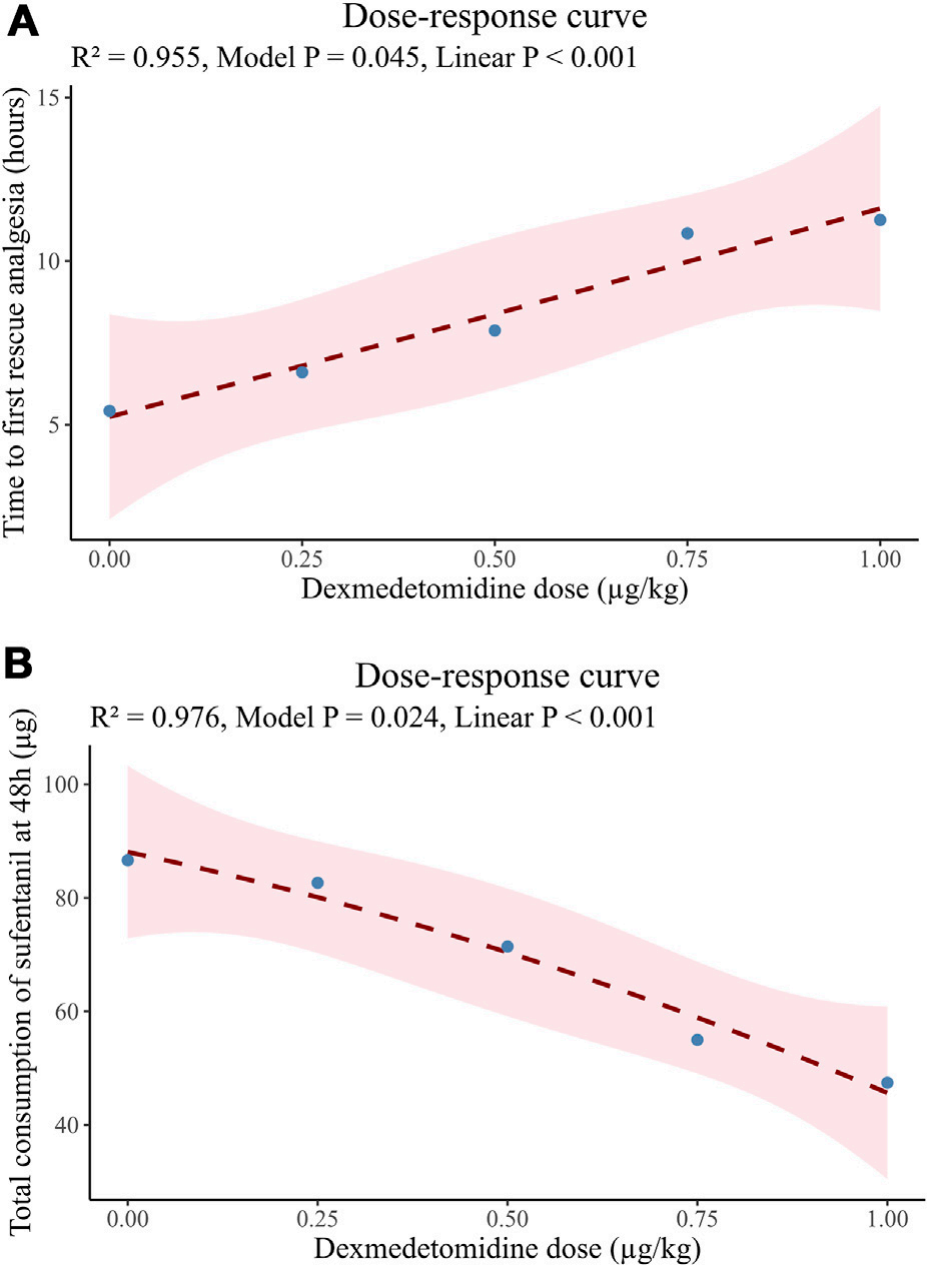
Dose-response curves for the primary outcomes. The time to first rescue analgesia **(A)** increased with higher doses of dexmedetomidine; the total consumption of sufentanil at 48 h **(B)** decreased as the dexmedetomidine dose increased.

**FIGURE 3:**
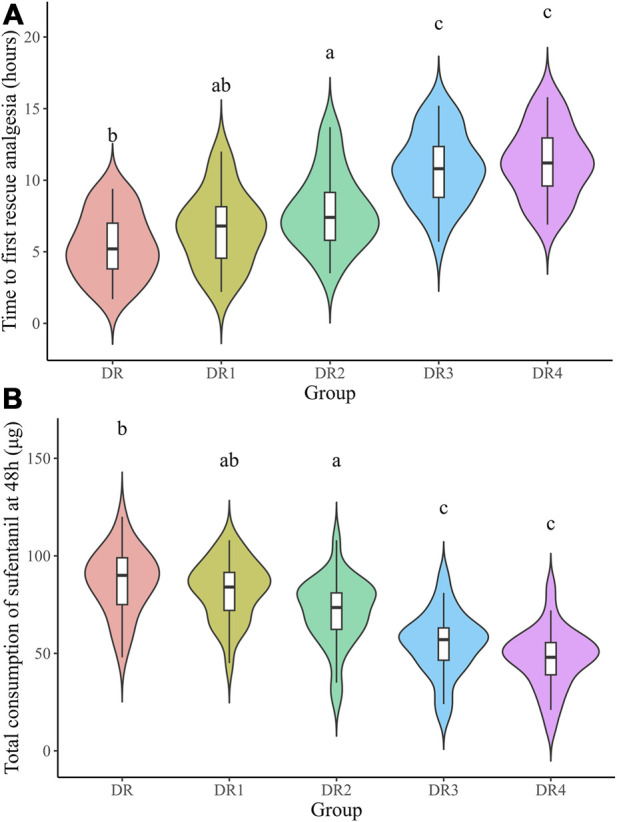
Violin and boxplots for time to first rescue analgesia **(A)** and total consumption of sufentanil at 48 h **(B)**. Violin plots illustrate the data distribution, and the overlaid boxplots display the median and interquartile range. Differences among groups were assessed using ANOVA, followed by Tukey’s HSD test for *post hoc* pairwise comparisons. Groups sharing at least one identical letter above the boxes were not significantly different, whereas groups with no common letter indicated statistically significant differences (Tukey-adjusted *P* < 0.05).

Trend analysis revealed a significant linear trend (*P* < 0.001) and a non-significant quadratic trend (*P* = 0.931), indicating that time to first rescue analgesia increased linearly with increasing doses of dexmedetomidine (Figure 2A). In addition, the violin and boxplots in Figure 3A illustrated the full distribution and comparative results across the five dose groups, further confirming a dose-dependent relationship between dexmedetomidine and time to first rescue analgesia.

### Total consumption of sufentanil at 48 h

The total consumption of sufentanil at 48 h was 86.62 ± 16.58 µg in group DR, 82.67 ± 15.08 µg in group DR1, 71.43 ± 17.28 µg in group DR2, 55.00 ± 16.30 µg in group DR3, and 47.44 ± 15.41 µg in group DR4. The overall difference among groups was also statistically significant (*P* < 0.001) (Table 2). Pairwise comparisons (Table 3) showed that compared with group DR, the mean difference in total consumption of sufentanil at 48 h was −15.19 µg (95% CI: −27.04 to −3.35; *P* = 0.005) for group DR2, -31.62 µg (95% CI: −43.58 to −19.66; *P* < 0.001) for group DR3, and -39.18 µg (95% CI: −51.13 to −27.22; *P* < 0.001) for group DR4. Compared with group DR1, the mean difference in total consumption of sufentanil at 48 h was −27.67 µg (95% CI: −39.83 to −15.50; *P* < 0.001) for group DR3 and -35.22 µg (95% CI: −47.39 to −23.05; *P* < 0.001) for group DR4. Compared with group DR2, the mean difference in total consumption of sufentanil at 48 h was −16.43 µg (95% CI: −28.49 to −4.37; *P* = 0.002) for group DR3 and -23.98 µg (95% CI: −36.04 to −11.93; *P* < 0.001) for group DR4. No statistically significant differences were found in the other comparisons.

Trend analysis similarly demonstrated a significant linear trend (*P* < 0.001) and a non-significant quadratic trend (*P* = 0.282), supporting a dose-response relationship in which total consumption of sufentanil at 48 h decreased linearly with increasing doses of dexmedetomidine (Figure 2B). In addition, the violin and boxplots in Figure 3B illustrated the full distribution and comparative results across the five dose groups, further confirming a dose-dependent relationship between dexmedetomidine and total consumption of sufentanil at 48 h.

### Secondary outcomes

**FIGURE 4:**
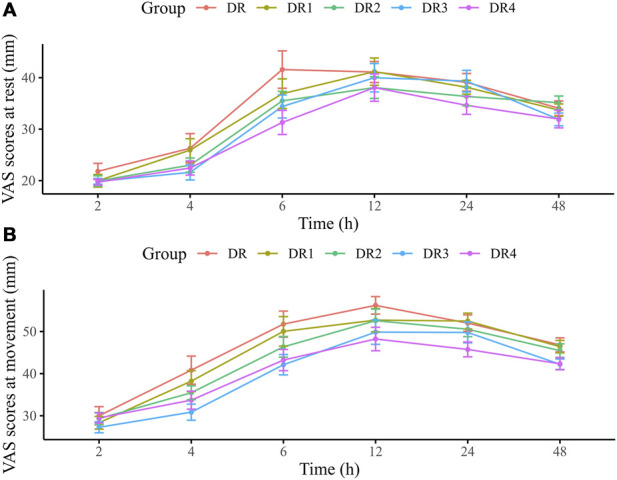
Line graphs of VAS scores at rest **(A)** and VAS scores with movement **(B)**. Data was presented as mean ± standard error for each cohort at each time point. The group × time interaction effects for VAS scores at rest and with movement were not significant. VAS = visual analogue scale.

In addition to evaluating the time to first rescue analgesia and total consumption of sufentanil at 48 h, we compared several other pain-related outcomes after CS. The results of the LMM analysis (Table 4) showed that the time effects of VAS scores at rest and with movement were statistically significant (*P* < 0.001). The group effect of VAS scores at rest was not statistically significant (*P* = 0.156), whereas the group effect of VAS scores with movement was statistically significant (*P* = 0.015). According to the *post hoc* analysis, groups DR3 (*P* = 0.037) and DR4 (*P* = 0.042) had significantly lower overall VAS scores with movement compared with group DR. However, the group × time interaction effects for VAS scores at rest (*P* = 0.620) and with movement (*P* = 0.641) were not significant, indicating that the trends in VAS scores over time were generally similar across the five groups, as also shown in the line graphs (Figure 4A for rest; Figure 4B for movement). Moreover, the time to first ambulation after surgery in group DR4 [24.00 h (22.95, 24.90)] was statistically significantly shorter than that in group DR [26.30 h (25.20, 27.20); *P* < 0.001], group DR1 [25.80 h (25.10, 27.00); *P* < 0.001], and group DR2 [25.60 h (25.08, 26.35); *P* = 0.002]. Similarly, group DR3 [24.30 h (22.80, 25.30)] showed a significantly earlier ambulation time compared with group DR (*P* < 0.001), group DR1 (*P* = 0.003), and group DR2 (*P* = 0.023). No statistically significant differences were found in the other comparisons (Table 5). Furthermore, no significant intergroup differences were found in the length of hospital stay and overall patient satisfaction scores (Table 5).

**TABLE 4:** Results of the linear mixed-effects model analyses for VAS scores.

Variable	Effect	NumDF	DenDF	F Value	P Value
VAS scores at rest	Group	4	133.00	1.69	0.156
	Time	5	665.00	112.70	<0.001
	Group: Time	20	665.00	0.87	0.620
VAS scores with movement	Group	4	133.00	3.20	0.015
	Time	5	665.00	119.09	<0.001
	Group: Time	20	665.00	0.86	0.641

DenDF, denominator degrees of freedom, NumDF, numerator degrees of freedom; VAS = visual analogue scale.

**TABLE 5:** Comparison results for other secondary outcomes.

Variable	Group DR (n = 29)	Group DR1 (n = 27)	Group DR2 (n = 28)	Group DR3 (n = 27)	Group DR4 (n = 27)	P Value
Time to first ambulation (hours)	26.30 (25.20, 27.20)^a^	25.80 (25.10, 27.00)^a^	25.60 (25.08, 26.35)^a^	24.30 (22.80, 25.30)^b^	24.00 (22.95, 24.90)^b^	<0.001
Length of hospital stay (hours)	91.90 (88.90, 93.50)	93.90 (91.00, 96.50)	93.20 (89.10, 95.05)	91.80 (87.75, 94.15)	90.90 (87.10, 93.00)	0.200
Patient satisfaction score	​	​	​	​	​	0.064
Very satisfied	3 (10.3)	2 (7.4)	4 (14.3)	7 (25.9)	10 (37.0)	​
Satisfied	12 (41.4)	15 (55.6)	18 (64.3)	14 (51.9)	12 (44.4)	​
Neutral	10 (34.5)	8 (29.6)	6 (21.4)	6 (22.2)	4 (14.8)	​
Unsatisfied	4 (13.8)	2 (7.4)	0 (0.0)	0 (0.0)	1 (3.7)	​
Very unsatisfied	0 (0.0)	0 (0.0)	0 (0.0)	0 (0.0)	0 (0.0)	​
Postoperative complications						
Hypotension	1 (3.4)	1 (3.7)	1 (3.6)	1 (3.7)	1 (3.7)	1.000
Sinus bradycardia	1 (3.4)^a^	1 (3.7)^ab^	3 (10.7)^ab^	6 (22.2)^ab^	10 (37.0)^b^	0.004
PONV	13 (44.8)^a^	9 (33.3)^ab^	5 (17.9)^ab^	2 (7.4)^b^	1 (3.7)^b^	0.001

Values were presented as median (interquartile range) or number (%).

Groups sharing the same superscript letter were not significantly different in *post hoc* pairwise comparisons (*P* ≥ 0.05); different letters indicated significant differences (*P* < 0.05).

PONV, postoperative nausea and vomiting.

**FIGURE 5:**
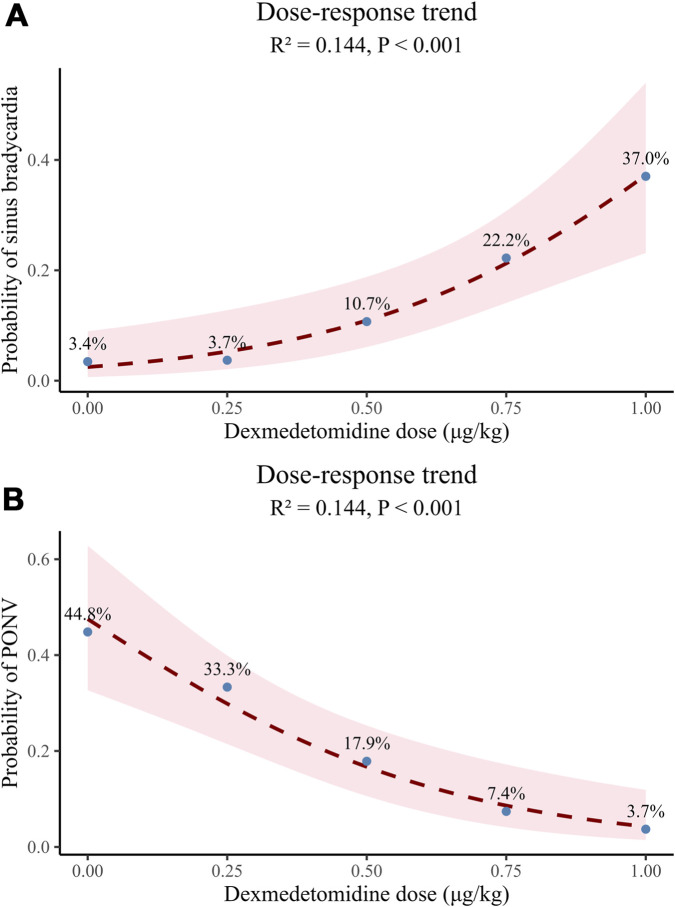
Dose-response trends for adverse events. As the dexmedetomidine dose increased, the incidence of sinus bradycardia **(A)** increased, whereas that of PONV **(B)** decreased. PONV = postoperative nausea and vomiting.

We investigated the side effects associated with dexmedetomidine including hypotension (5 cases, 3.6%), sinus bradycardia (21 cases, 15.2%), PONV (30 cases, 21.7%), and pruritus (1 case, 0.7%). The study cohort demonstrated zero incidence of hypoxemia, respiratory depression, and neurotoxicity. We found no differences in hypotension between any groups (Table 5). At the 48 h, the incidence rates of sinus bradycardia among the five groups were as follows: group DR (3.4%), group DR1 (3.7%), group DR2 (10.7%), group DR3 (22.2%), and group DR4 (37.0%). A statistically significant difference was observed only between groups DR and DR4 (*P* = 0.025), whereas no differences were found in other intergroup comparisons (Table 5). And at the 48 h, the incidence rates of PONV among the five groups were as follows: group DR (44.8%), group DR1 (33.3%), group DR2 (17.9%), group DR3 (7.4%), and group DR4 (3.7%). A statistically significant difference was observed between groups DR and DR3 (*P* = 0.035) as well as between groups DR and DR4 (*P* = 0.010), whereas no significant differences were found among the other intergroup comparisons (Table 5). In addition, Logistic regression analysis showed that, with increasing doses of dexmedetomidine, the incidence of sinus bradycardia (Figure 5A) increased, while the incidence of PONV (Figure 5B) decreased. All documented adverse events were asymptomatic and required no clinical intervention.

## Discussion

In this randomized, double-blind, controlled clinical trial, we evaluated the safety and efficacy of different doses of dexmedetomidine as adjuvants to ultrasound-guided TAP block for postoperative analgesia after CS. The primary results demonstrated that with increasing doses of dexmedetomidine, the time to first rescue analgesia was significantly prolonged, and the total consumption of sufentanil at 48 h was markedly reduced. However, the higher dose of 1.00 μg/kg offered no significant benefit over 0.75 μg/kg. The higher-dose groups (0.75 and 1.00 μg/kg) were associated with improved overall VAS scores with movement, earlier time to first ambulation, and a reduced incidence of PONV, with no significant differences between the two doses. Notably, although the incidence of sinus bradycardia increased in a dose-dependent manner, a statistically significant increase was observed only in 1.00 μg/kg group compared with control group. No significant differences were observed among the five groups in VAS scores (group × time interaction effects), length of hospital stay, incidence of hypotension, or overall patient satisfaction scores. Taken together, dexmedetomidine at 0.75 μg/kg appeared to achieve most favorable balance between prolonged analgesia, reduced opioid consumption, earlier postoperative ambulation, and a lower risk of adverse reactions, and could represent the ideal dose for ultrasound-guided TAP block with ropivacaine in CS.

Dexmedetomidine is a potential anesthetic adjuvant that can promote better postoperative analgesia, reduce postoperative analgesic requirements, and prolong the local anesthetic effect when administered in TAP block (Sun et al., 2019). However, since dexmedetomidine may cause side effects such as bradycardia, hypotension, and excessive sedation, the dose that provides effective analgesic with minimal side effects is controversial. 2.00 μg/kg of dexmedetomidine has been reported to reduce the minimum local anesthetic concentration used for TAP block and improve postoperative analgesia in children undergoing surgery for inguinal hernia repair or hydrocelectomy (Raof et al., 2017). A separate study suggested that TAP block using 0.50, 1.00, and 2.00 μg/kg dexmedetomidine combined with ropivacaine was effective treatment for analgesia in laparotomy procedures for gynecologic malignancies (Zeng et al., 2020). In patients receiving TAP block for CS, the addition of 0.50 μg/kg dexmedetomidine to ropivacaine prolonged the duration of analgesia (Qian et al., 2020). In addition, multiple studies have shown that the addition of 1.00 μg/kg dexmedetomidine to ropivacaine for ultrasound-guided TAP block significantly prolonged postoperative analgesia duration following CS (Singla et al., 2021; Nag et al., 2024). However, with the increase of dexmedetomidine dosage (for example, 1.00, 1.50, and 2.00 μg/kg), no clinically meaningful enhancement in analgesic efficacy was observed, and some series of related side effects could inevitably occur (Zeng et al., 2020). Therefore, this study selected 0.00, 0.25, 0.50, 0.75, and 1.00 μg/kg of dexmedetomidine for further investigation in order to explore a potential lower effective analgesic dose. In this trial, we found that both 0.75 and 1.00 μg/kg of dexmedetomidine were superior to the lower doses in prolonging analgesia, reducing opioid consumption, promoting earlier postoperative ambulation, and decreasing the incidence of PONV. Furthermore, the 0.75 μg/kg dose showed no significant difference from the 1.00 μg/kg dose in these outcomes. Notably, the incidence of sinus bradycardia increased significantly with higher dexmedetomidine doses, reaching 37.0% in group DR4. These results suggested that 0.75 μg/kg of dexmedetomidine could be a choice in clinical practice, providing sufficient analgesic efficacy while reducing the adverse effects associated with higher doses.

In a previous study, when 1.0 μg/kg dexmedetomidine was used as an adjuvant for TAP block after CS, the analgesic duration was 18.21 ± 3.14 h, compared with 11.26 ± 2.48 h in our study (Nag et al., 2024). This difference may be attributed to the different concentrations of ropivacaine used: Nag et al. administered 40 mL of 0.75% ropivacaine, whereas we used 40 mL of 0.25% ropivacaine. In another trial utilizing 40 mL of 0.3% ropivacaine combined with 1.0 μg/kg dexmedetomidine for TAP block, the time to first rescue analgesia was 682.43 min (Zeng et al., 2020), which is similar to our results. In addition, the results of Singla et al. are comparable to our study, further indicating that the reason for this difference may be related to the concentration of ropivacaine used for TAP block (Singla et al., 2021). Ropivacaine, a member of the amino amide class of local anesthetics, is used as the local anesthetic for TAP block in the current study as it has higher safety margin and lower levels of cardiac toxicity compared to bupivacaine (Finucane, 2005). Previous studies have shown that 0.25%–0.50% ropivacaine for TAP block is effective (De Oliveira et al., 2011; Abdul Jalil et al., 2014; Xue et al., 2022). In addition, considering the anthropometric characteristics of our regional population, where pregnant women exhibited a BMI of 25.90 ± 2.30 kg/m^2^, we used 0.25% ropivacaine to ensure clinical safety while maintaining analgesic efficacy.

In this study, sufentanil consumption was significantly reduced, whereas VAS pain scores showed no significant differences among groups. This reflects the synergistic analgesic effects of TAP block and dexmedetomidine. On the one hand, TAP block directly blocks peripheral nociceptive signal transmission, producing local analgesia. On the other hand, dexmedetomidine not only prolongs the analgesic duration of TAP block via vasoconstriction and action potential suppression, but also exerts intrinsic analgesic effects through activation of central and peripheral α_2_ receptors and modulation of inflammatory pathways (Bao et al., 2022; Liao et al., 2025). Together, these mechanisms produce a substantial opioid sparing effect. Consequently, despite reduced opioid consumption, maternal pain levels were maintained at comparable levels, which reflects the success of this analgesic strategy rather than inadequate analgesia. In addition, this phenomenon may be partly attributed to a floor effect of VAS scores under effective analgesia, as well as inherent limitations of the VAS measurement. Beyond preserved analgesic efficacy, the marked reduction in opioid consumption directly attenuated opioid induced nausea and vomiting. Furthermore, dexmedetomidine may independently reduce the incidence of PONV by decreasing sympathetic outflow and catecholamine release (Zhao et al., 2023). In summary, using dexmedetomidine as an adjuvant in TAP block achieves effective opioid sparing and PONV reduction while preserving analgesic quality, fully aligning with multimodal analgesia principles.

The common adverse events of dexmedetomidine are hypotension and sinus bradycardia. However, in this study, there was no statistically significant difference in the incidence of hypotension among the five groups. Regarding sinus bradycardia, our study found that the incidence of sinus bradycardia increased significantly with increasing doses of dexmedetomidine, reaching 37.0% in the 1.00 μg/kg group. A significant difference was observed between the 0.00 and 1.00 μg/kg groups, whereas no statistically significant differences were found among the other intergroup comparisons. These results differ from those of a previous report and may be attributable to the fact that the TAP block in our study was performed immediately after CS (Jung et al., 2018). Additionally, the relatively high incidence of sinus bradycardia observed in this study may be related to the intraoperative use of phenylephrine to treat hypotension following CSEA and to the potentially high sensitivity of the study population to dexmedetomidine. Although these adverse events were all mild and required no intervention, they still indicated a higher risk of cardiac side effects with high-dose dexmedetomidine. Therefore, the dose of 0.75 μg/kg dexmedetomidine could present most favorable balance between prolonging the analgesic effect and reducing the incidence of adverse effects.

There were some limitations to our study. First, if the mother was concerned that opioids might affect herself or her infant, this concern could have influenced the time to first rescue analgesia and amount of opioid consumption. Second, because the TAP block was administered under CSEA, it was impossible to determine whether the TAP block was successful, and the exact duration of sensory blockade could not be objectively assessed. Third, plasma concentrations of dexmedetomidine were not measured, and the long-term neurotoxic profile of perineural dexmedetomidine remains incompletely characterized. These important issues warrant careful consideration in future studies. Finally, the absence of non-opioid analgesics such as paracetamol or non-steroidal anti-inflammatory drugs may limit the generalizability of our findings to standard multimodal analgesia strategy. Nevertheless, our results demonstrate the synergistic analgesic effect of perineural dexmedetomidine in TAP block, which may provide a useful basis for future multimodal regimen refinement.

## Conclusion

In conclusion, dexmedetomidine as an adjuvant to ropivacaine in ultrasound-guided TAP block for analgesia after CS demonstrated a clear dose-dependent effect. With increasing doses, the duration of analgesia was prolonged, opioid consumption decreased, and early postoperative recovery was facilitated. However, a significantly increased risk of sinus bradycardia was associated with higher doses. Considering both analgesic efficacy and safety, 0.75 μg/kg dexmedetomidine provided most favorable balance, offering prolonged analgesia, reduced opioid requirements, and facilitated early recovery while maintaining a lower rate of adverse reactions. Further studies are needed to validate the applicability and long-term safety of this dosage in different populations and surgical settings.

## Data Availability

The raw data supporting the conclusions of this article will be made available by the authors, without undue reservation.
